# Adoption of mobile health services using the unified theory of acceptance and use of technology model: Self-efficacy and privacy concerns

**DOI:** 10.3389/fpsyg.2022.944976

**Published:** 2022-08-11

**Authors:** Yizhi Liu, Xuan Lu, Gang Zhao, Chengjiang Li, Junyi Shi

**Affiliations:** ^1^College of Management, Guizhou University, Guiyang, China; ^2^School of Engineering, University of Tasmania, Hobart, TAS, Australia; ^3^School of Humanities and Social Sciences, Xi’an Jiaotong-Liverpool University, Suzhou, China

**Keywords:** self-efficacy, privacy concerns, UTAUT model, mHealth services, intention to adopt

## Abstract

Mobile health (mHealth) services have been widely used in medical services and health management through mobile devices and multiple channels, such as smartphones, wearable equipment, healthcare applications (Apps), and medical platforms. However, the number of the users who are currently receiving the mHealth services is small. In China, more than 70% of internet users have never used mHealth services. Such imbalanced situation could be attributed to users’ traditional concept of medical treatment, psychological factors (such as low self-efficacy) and privacy concerns. The purpose of this study is to explore the direct and indirect effects of mHealth users’ self-efficacy and privacy concerns on their intention to adopt mHealth services, providing guidelines for mHealth service providers to enhance users’ intention of adoption. A questionnaire was designed by the research team and 386 valid responses were collected from domestic participants in China. Based on the unified theory of acceptance and use of technology (UTAUT) model, a research model integrated self-efficacy and privacy concerns was constructed to investigate their effects on users’ intention to adopt mobile mHealth services. The results show that self-efficacy could facilitate users’ intention to adopt mHealth services, and had a significantly positive effect on perceived ubiquity, effort expectancy, performance expectancy and subjective norm. This study verifies the direct and indirect effects of self-efficacy and privacy concerns on users’ intention to adopt mHealth services, providing a different perspective for studying mHealth adoption behavior. The findings could provide guidelines for mHealth service providers to improve their service quality and enhance users’ intention of adoption.

## Introduction

Mobile health (mHealth) services refer to the provision of medical services to users through mobile devices, such as smartphones, tablet computers, and satellite communications (Jovanov and Zhang, 2004; Bai et al., 2020; Bally and Cesuroglu, 2020; Lee, 2021; Sujarwoto et al., 2022). mHealth services have changed the traditional healthcare and played an increasing important role in the medical service delivery through their unique features, such as ease of use, usefulness and convenience (Liu et al., 2019; Crowell et al., 2022; Zhao et al., 2022). More and more patients are obtaining medical information and services through mobile devices, including making appointment, seeking treatment, viewing electronic test reports and consulting the doctors (Lee and Han, 2015; Wang and Qi, 2021; Balica, 2022; Jenkins, 2022), which effectively alleviates the problem of the “queuing for 3 h but seeing the doctor for 3 m” in China. mHealth services have become a new approach for people’s health management (Park, 2016).

With the integration and development of “Internet + medical healthcare,” mHealth service users refer to people who obtain mHealth services through channels such as hospital’s official website, Weibo (a platform for information sharing, dissemination, commenting and acquisition based on user relationship; Zhang and Pentina, 2012), WeChat public account (a platform for interaction and communication between the host and subscribers; Geng, 2016), WeChat mini program (a fast App with click-to-use function and free download and installation; Cheng et al., 2019), mHealth service App, and provincial and municipal medical platforms. By December 2020, there were 989 million netizens in China, but only 215 million mHealth service users. That means more than 70% of netizens have never used mHealth services. In addition, the users’ intention of continuous use of mHealth services is not high (Serlachius et al., 2019) due to the influence of traditional concept of medical treatment, users’ psychological factors and privacy concerns considering that mHealth is an innovative App of information technology in the medical field (Hsiao and Tang, 2015; Zhou et al., 2019). Therefore, how to motivate and guide users to use mHealth services and meet their medical needs more effectively has become an urgent issue for mHealth providers.

Existing studies have explored the influential factors and mechanisms of users’ adoption behavior of mHealth services based on different theoretical models. Many scholars have mainly focused on technical features of mHealth services (AlBar and Hoque, 2019; Nadal et al., 2020; Nezamdoust et al., 2022), App platform design (Miao et al., 2018; Wu et al., 2020) or factors related to the external environment which influence users’ adoption and continuous use of mHealth services (Li et al., 2016; Chen et al., 2018). Less attention has been paid to the individual cognitive factors that affect users’ adoption of mHealth services.

According to previous studies, in the context of mobile healthcare, the individual cognitive factors that affect users’ adoption behavior mainly include attitude, trust, intrinsic motivation, self-efficacy, privacy concerns, IT personal innovativeness, mobile technology identity, technology anxiety, and electronic health knowledge (Andronie et al., 2021b, pp. 863–888). These individual cognitive factors have been used in numerous studies to explain the decision process of mHealth adoption behavior (Andronie et al., 2021a, pp. 2497; Lãzãroiu et al., 2022). For example, Deng et al. (2018) identified trust and privacy risk as the most dominant individual cognitive factors and critical factors explaining Chinese patients’ behavioral intention on mHealth. Rajak and Shaw (2021) showed that technology anxiety negatively affected perceived usefulness and perceived ease of use, while data privacy might be a cause of technology anxiety. In addition, self-efficacy was acknowledged to play a crucial role in influencing users’ adoption behavior towards mHealth (Zhang et al., 2017). Alam et al. (2020) found that self-efficacy was positively associated with mHealth adoption intentions.

In users’ acceptance and use of mHealth services, self-efficacy and privacy concerns are critical to adoption behaviors because users may refuse to accept or use mHealth services when users believe that they are incapable to access medical services through mobile devices, or fear that personal privacy information will be leaked without authorization. In this study, self-efficacy refers to the user’s self-perception about whether they will be capable of using mHealth services, while privacy concerns refer to the level of the users’ anxiety about how the mHealth App collect, utilize and protect their personal information. Balapour et al. (2019) conducted an innovative study on patients’ self-monitoring health using mHealth Apps, confirming that self-efficacy promoted patients’ healthy behaviors and the use of mHealth Apps. Li (2020) studied the adoption and use of mHealth services by Chinese users, pointing out that privacy concerns were an important factor hindering users from adopting mHealth services. However, the limitations of these studies are that they did not consider the influence of self-efficacy and privacy concerns, the two typical individual cognitive factors, on users’ adoption behavior of mHealth services at the same time.

mHealth is an emerging form of healthcare with mobile technology in the medical field. Users are the main body of the adoption of mHealth services. However, existing studies did not pay enough attention to the psychological factors of individuals, ignoring the influence of users’ self-efficacy and privacy concerns on their intentions to adopt mHealth services. To better understand and predict the adoption behavior of mHealth users, based on the unified theory of acceptance and use of technology (UTAUT) model, this study introduces two individual cognition factors, self-efficacy and privacy concerns into the model and investigates their effects on users’ intention to adopt mHealth services. Therefore, this paper aims to answer the following research questions:

(1) How do mHealth users’ intention of adoption arise? What are the individual cognitive factors that influence users’ intention to adopt?
(2) What is the role of users’ self-efficacy, privacy concerns, and UTAUT-related variables in the decision process of mHealth users’ intention to adopt?

Compared with existing studies, this study focuses on user’s individual cognition and uses UTAUT theory to analyze the influence of self-efficacy and privacy concerns on the intention to adopt mHealth. By adding two representative individual cognitive factors (self-efficacy and privacy concerns) into the UTAUT model, we can predict and explain users’ mHealth adoption behavior more comprehensively and provide insights for mHealth adoption behavior research. Meanwhile, exploring the four perceptual factors of UTAUT as mediating variables helps better the interpret UTAUT in mHealth and also provides new ideas to extend the App of the UTAUT model in mHealth services. Finally, the findings of this paper help mHealth service providers understand the effect of individual cognitive factors on users’ intention to adopt, and provide theoretical basis and pragmatic suggestions for improving their healthcare services and enhancing users’ intention to adopt.

The remaining chapters of this study are organized as follows: section “Literature review and hypotheses development” provides the theoretical background. Section “Research methodology” presents the hypotheses. Section “Results” describes the research design, and section “Discussion” discusses the results. Section “Conclusion” is the conclusion of the study.

## Literature review and hypotheses development

Existing literature related to mobile health mainly focuses on the definition of mobile healthcare, service types, stakeholders, and user adoption behaviors. Istepanian et al. (2007) first proposed the concept of “mobile healthcare,” and defined it as a medical system that used mobile communication and network equipment to provide health services. Motamarri et al. (2014) classified the mHealth services into medical information service, diagnosis service, disease monitoring service, health data monitoring service and telemedical service. Meanwhile, Dehzad et al. (2014) categorized mHealth stakeholders as policy makers, users, service providers and other stakeholders. In addition, regarding the adoption behavior of mHelath users, existing studies have explored the influential factors of users’ adoption behavior based on different theoretical models, as shown in Table 1. Garavand et al. (2019) studied the influencing factors of medical students’ adoption of mHealth services based on the UTAUT model, and pointed out that performance expectancy and social influence were not significantly related to adoption behavior. Deng et al. (2018) considered the influence of trust and perceived risk on users’ intention to adopt based on the technology acceptance model (TAM), and the results showed that trust, perceived usefulness and perceived ease of use had a significant positive impact on intention of adoption.

**TABLE 1:** Research related to mHealth adoption intentions.

Type	Independent variable	Model	References
mHealth User	Trust, Perceived risk Perceived ease of use, Perceived usefulness	TAM	Deng et al., 2013
Seniors	Perceived usefulness, Perceived ease of use Technology anxiety, Refusal to change	TAM, Two-factor model	Guo et al., 2015
Medical Practitioners	Perceived usefulness, Perceived ease of use, Subjective norm, Attitudes	TAM, TPB	Wu et al., 2020
mHealth Potential User	Privacy concerns, Perceived personalization, Trust	The Privacy-personalization paradox	Guo et al., 2016
Medical Students	Effort expectancy, Performance expectancy, Social influence, Facilitating conditions	UTAUT, TRA	Garavand et al., 2019

In addition, many studies have confirmed that some main individual cognition factors, such as self-efficacy, privacy concerns, perceived risk, and technology anxiety, have a direct or indirect impact on the adoption behavior of mHealth users. Pan et al. (2019) conducted a study on the adoption intentions of clinicians and non-clinicians from the perspective of technology transfer, and confirmed that subjective norm had a positive effect on clinicians’ behavioral intention, while perceived risk had a negative effect only on non-clinicians’ attitude. Based on protection motivation theory (PMT), Guo et al. (2015) explored the influence of threat appraisal and coping appraisal on the adoption intention of mHealth users. The results showed that users’ threat appraisal and coping appraisal negatively influenced the intention of adoption through attitude. At the same time, privacy concerns are regarded as one of the risks for users to adopt mHealth services. Guo et al. (2016), based on the privacy personalization paradox, pointed out that privacy concerns had a significant negative impact on users’ intention to adopt mHealth services. It is worth noting that some studies segment users according to different service types, which often leads to inconsistent conclusions. For example, Meng et al. (2020), explored the negative impact of elderly users’ characteristics (health anxiety and technology anxiety) on intention of continuance of use based on trust theory. Lim et al. (2011) studied Singaporean women’s acceptance of using mobile phones to seek health information and found that technology anxiety had no significant effect on female users’ intention to use. In addition, Deng (2013) pointed out that the perceived ease of use had no significant effect on the behavioral attitude of elderly users, while the empirical results of Hsiao and Chen (2015) showed that the perceived ease of use had significant effects.

### Unified theory of acceptance and use of technology

Mobile health is an innovative App of mobile technology in healthcare, and the study of mobile health users’ intention to adopt is a part of the study of information technology adoption behavior. The TAM is used as a common model of user adoption behavior for new technologies, explaining how perceived usefulness and ease of use affect the user adoption decision process. On the basis of TAM, Venkatesh et al. (2003) proposed the UTAUT, in which users’ behavioral intentions are influenced by effort expectancy, performance expectancy, social influence and facilitating conditions. Alaiad et al. (2019) argued that the UTAUT model could reflect about 70 percent of the variables in users’ intention of adoption. To enhance the understanding of the extent of technology adoption in the UTAUT model, Venkatesh et al. (2012) proposed UTAUT2 with the addition of variables of hedonic motivation, price value, and habit. With the emerging technologies, the UTAUT model has been frequently applied to users’ adoption behaviors in various health-related studies, including information systems (Hadji and Degouelt, 2016), medical institutions (Sun and Rau, 2015), and mobile healthcare (Nisha et al., 2019) and telehealth care services (Park, 2009).

In the context of mHealth services, Shiferaw and Mehari (2019) introduced self-efficacy into the research on the acceptance behavior of electronic medical record systems by doctors and nurses based on the UTAUT model, and found that self-efficacy, effort expectancy, performance expectancy, social influence and facilitating conditions significantly and positively affected the actual usage behavior of users. Similarly, Nisha et al. (2019) chose the UTAUT model as their theoretical framework to explore the factors that affected the users’ intention of adoption of mHealth in Bangladesh. The results showed that new users were particularly sensitive to effort expectancy, performance expectancy, social influence, facilitating conditions and trust, which could significantly promote the usage intention of mHealth. In addition, based on PMT and UTAUT model, Hsieh et al. (2017) showed that perceived ease of use, self-efficacy and perceived usefulness were the important factors that affected behavioral intention of personal health record.

Although TAM, UTAUT and their extended models are useful, many efforts have been made to improve their explanatory power. The Theory of Reasoned Action (TRA) has proven to be one of the effective models to explain users’ behavioral intentions (Fishbein and Ajzen, 1975). Individuals are always rational in the behavioral decision making, and the actual behavior made by users is mainly influenced by behavior intention, while attitude toward behavior and subjective norm are two important factors that influence users’ intention to adopt. Subjective norm refers to the pressure exerted by people or organizations that have a significant influence on the user when making behavioral decisions and is equivalent to the variable of social influence in UTAUT (Taylor and Todd, 1995a). When the user’s attitude toward a behavior is positive and the person with significant influence also suggests the user to adopt the behavior, it will reinforce the user to produce the actual usage behavior. In this study, subjective norm from the TRA was selected as an individual cognitive factor to explore the decision process of users’ intention to adopt.

The Health Belief Model (HBM) has been widely used in the study of user information technology adoption behavior and proven as an effective model to explain behavioral intention. According to the HBM, human behavior is determined by health beliefs, behavioral cues or intentions, and constraints on behavior. Kim and Park (2012) constructed mHealth users’ adoption model from three dimensions: health, information and technology, based on TAM and Health belief model. Comparing TAM and HBM predictions of user behavioral intention, Mathieson (1991) concluded that both theories were appropriate. The former model is easy to be applied, while the latter can capture most aspects of an individual’s beliefs through a large number of variables (Chuttur, 2009).

With the development of mHealth services, privacy concerns are gaining attraction from researchers. In mHealth services contexts, users have relatively limited control over health data collection and usage, and are therefore more likely to suffer losses from privacy breaches. The privacy-personalization paradox has been developed to explain users’ behavioral intentions. Users’ desire for a wide range of personalized services requires service providers to collect more personal information, yet users are reluctant to disclose, leading to an apparent technological paradox (Awad and Krishnan, 2006). Milholland (1994) argued that the electronic storage of personal information and medical records posed a threat to user privacy. This threat is exacerbated by mHealth Apps. Privacy concern is defined as the extent to which individuals are concerned about the security of their privacy information (Cocosila and Archer, 2010). Based on the privacy-personalization paradox, Guo et al. (2016) constructed an adoption intention model including privacy concern, perceived personalization and trust, and the findings showed that privacy concern had a significant negative impact on the intention of adoption.

**FIGURE 1:**
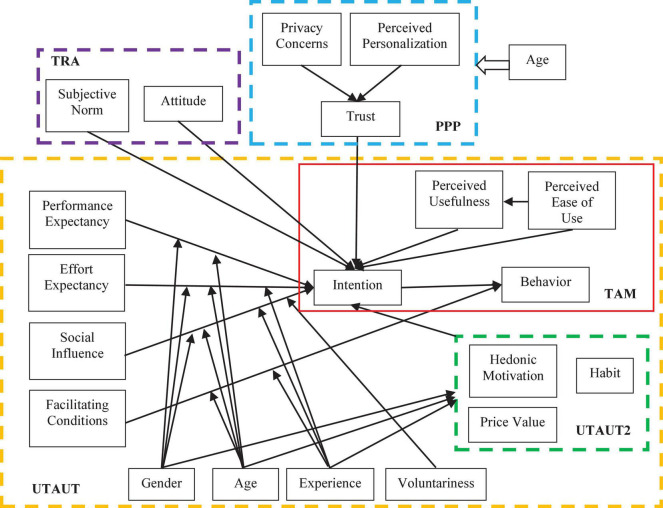
Unified theory of acceptance and use of technology (UTAUT) and its extended model.

Previous studies on mHealth user adoption behavior based on TAM, UTAUT, TRA related or independent conceptual models are summarized in Figure 1. In this study, based on the UTAUT model, we replace social influence in UTAUT by subjective norm. Ajzen (1991) argued that subjective norm was an important factor to explain and predict the user’s usage behavior of information system. Meanwhile, perceived ubiquity is proposed in the field of information technology and is similar to perceived mobility of services, i.e., the extent to which users can access information or services anytime and anywhere (Amberg et al., 2004). Compared to traditional access channels, the most important feature of mHealth is the ubiquity of services that enables users to access mHealth services anytime, anywhere in any situation. We replace facilitating conditions in UTAUT by perceived ubiquity, which is more in line with the significant features that influence potential users’ adoption behavior in the mobile technology context. Extending the UTAUT model (i.e., effort expectancy, performance expectancy, subjective norm, and perceived ubiquity) by introducing two prediction variables of self-efficacy and privacy concerns in, we comprehensively examine the effect of self-efficacy and privacy concerns on the adoption of mHealth services in China.

### Effort expectancy

Effort expectancy refers to the level of effort that users believe is required to use the new technology. Studies have confirmed that effort expectancy is equivalent to perceived ease of use in TAM (Wu and Wang, 2005; Schaper and Pervan, 2007), and the effort expectancy of different user groups varies. Effort expectancy has a significant positive effect on users’ behavioral intention (Kim et al., 2015; Duarte and Pinho, 2019). Regarding mHealth services, if users perceive the simpler use or operation of mHealth services is and the less effort (including time, energy, etc.) they need to make, the stronger their intention to adopt mHealth services will be. In summary, this study proposes the following hypothesis:

Hypothesis 1: Effort expectancy has a positive effect on users’ intention to adopt mHealth services.

### Performance expectancy

Performance expectancy refers to the user’s judgment that the mHealth services are beneficial to him. Many studies have shown that performance expectancy can directly and significantly affect users’ intention to adopt (Wu et al., 2011; Ifinedo, 2012). In the UTAUT model, Venkatesh et al. (2003) proposed and confirmed that performance expectancy had a significant positive effect on individuals’ behavioral intentions. Semiz and Semiz (2021) also reported that performance expectancy had an important impact on users’ intention to use mHealth Apps in their survey. Mobile healthcare can provide users with timely and valuable information resources, which can significantly reduce the time for users to queue for registration, saving much time, energy and physical costs. Therefore, we speculate that the higher the users perceive the usefulness of mHealth, the stronger their intention to adopt mHealth will be. In summary, this study proposes the following hypothesis:

Hypothesis 2: Performance expectancy has a positive effect on users’ intention to adopt mHealth services.

### Subjective norm

In this study, subjective norm refers to the degree to which users perceive those important persons who want or do not want them to use mHealth, similar to the social influence in UTAUT (Taylor and Todd, 1995b). Ajzen (1991) argued that subjective norm was an important factor to explain and predict the user’s usage behavior of information system. mHealth is an innovative App of “mobile technology + medical service.” Therefore, when users decide whether to adopt mHealth services, they often make decisions following others’ advices. In fact, the particularity of mHealth services makes subjective norm play a very important role in users’ intention of adoption. When important persons (relatives, friends, experts) recommend them to use mHealth services, their trust in products or services will be significantly improved, and the intention to use mHealth will be stronger (Semiz and Semiz, 2021). In summary, this study proposes the following hypothesis:

Hypothesis 3: Subjective norm has a positive effect on users’ intention to adopt mHealth services.

### Perceived ubiquity

Service ubiquity is a distinguishing feature of mobile technology, similar to the facilitating conditions in UTAUT (Okazaki and Mendez, 2013; Hsiao and Chen, 2015). Qian and Niu (2019) showed that the perceived ubiquity of mobile payment business had a positive influence on Alipay user adoption. The ubiquity of mHealth services is reflected in the possibilities for users to making appointment, seeking treatment, and inquiring about medical information or services anytime, anywhere, which brings users more freedom and convenience and effectively improve the efficiency of medical services. The higher the user’s perception of the ubiquity of mHealth services is, the higher satisfaction the users gain from their continuous use of mHealth services (Sneha and Varshney, 2009). At the same time, based on a meta-analysis, Zhu et al. (2020) also confirmed that perceived ubiquity had a significant positive effect on users’ intention to adopt mHealth. Their results showed that the feature that mobile healthcare was not limited by time, space and situation greatly improved the healthcare efficiency and users’ intention to adopt. Consequently, this study proposes the following hypothesis:

Hypothesis 4: Perceived ubiquity has a positive effect on users’ intention to adopt mHealth services.

### Self-efficacy

Self-efficacy refers to a user’s self-perception of his/her own abilities, essentially an individual’s subjective judgment, which can significantly affect the user’s intention to adopt (Balapour et al., 2019; Shiferaw and Mehari, 2019). Gagnon et al. (2014) found that when users were confident in their ability to use new technologies, they felt that the operation was easier. In the Chinese domestic healthcare industry, self-efficacy has a significant positive influence on users’ effort expectancy and performance expectancy (Zhu and Liu, 2016). Users with low self-efficacy will think that the operation of mHealth is more complicated, so they are less likely to use the services. When users believe that they have sufficient ability to use mobile technology, they will have a positive opinion of mobile healthcare, and believe that the use of mHealth services has brought convenience for them. Therefore, this study proposes the following hypotheses:

Hypothesis 5: User’s self-efficacy has a positive effect on effort expectancy.

Hypothesis 6: User’s self-efficacy has a positive effect on performance expectancy.

In the context of mHealth services, self-efficacy is an important factor affecting users’ intention to adopt (Wu et al., 2007; Weimer et al., 2017; Chao, 2019). Specifically, self-efficacy has a positive effect on subjective norm (Cho et al., 2015). If users have high self-efficacy and believe that they can learn or have the skills to use mHealth proficiently, this confidence may strengthen the willingness of people around them to use mHealth services. On the contrary, when the users’ self-efficacy is low, they may reduce the use of mHealth services to a certain extent, and even negatively affect the adoption behavior of the surrounding people. The stronger the sense of self-efficacy, the stronger the user’s confidence in using mHealth services, which leads to stronger subjective norm. Thus, this study proposes the following hypothesis:

Hypothesis 7: User’s self-efficacy has a positive effect on subjective norm.

Meanwhile, self-efficacy also influences perceived ubiquity to a certain degree. Studies have shown that the higher the user’s self-efficacy in the medical field, the stronger their perception of the service’s ubiquity, and the more positive their attitude toward mHealth services (Jian et al., 2012; Vanneste et al., 2013). In other words, when users have a high level of self-efficacy and are confident that they can obtain mHealth services by completing the operation process, they are more likely to accept or adopt mHealth services, and then believe that mHealth services are ubiquitous. Therefore, this study believes that when users have high perception of their ability or confidence in using mHealth service, the more ubiquitous they perceive mHealth services in their lives. Based on this claim, this study proposes the following hypothesis:

Hypothesis 8: User’s self-efficacy has a positive effect on perceived ubiquity.

In the study of information technology adoption, self-efficacy is often reported to have an effect on the intention of adoption as a user’s individual cognitive factor. Users with low self-efficacy are less likely to adopt a certain behavior (Bandura, 1986). Hsu and Chiu (2004) illustrated that self-efficacy was an important factor to explain consumers’ usage decisions in e-commerce, and confirmed that users’ self-efficacy had a positive influence on usage intention. Zhang et al. (2017) explored the influential factors of adoption behavior of mHealth service users. The results showed that self-efficacy played an important role in users’ intention of adoption. Fox and Connolly (2018) also found that the self-efficacy had a significant positive effect on intention to adopt mHealth services. Consequently, this study proposes the following hypothesis:

Hypothesis 9: User’s self-efficacy has a positive effect on intention to adopt mHealth services.

### Privacy concerns

Privacy concerns are defined as the degree to which users are concerned about the disclosure of personal information (Cocosila and Archer, 2010). In the context of mHealth services, when users do not know about or are not familiar with the services, they are afraid of leakage or abuse of their own privacy, especially if their information is used for other purposes without authorization, resulting in privacy leakage, property loss and other adverse consequences (Zhu et al., 2020). Many mHealth services involve personal health data and are carried out in a virtual network environment. Users’ privacy concerns will weaken their perception of the ease of use of the services, that is, users’ concerns about privacy will significantly reduce their effort expectancy. Therefore, this study proposes the following hypothesis:

Hypothesis 10: Users’ privacy concerns negatively influence effort expectancy.

Furthermore, health data are regarded as absolutely private information (Matteo et al., 2018), and users’ concerns about health data security and privacy are one of the reasons why they do not adopt or continue to use mHealth services (Wang and Qi, 2021). As mobile healthcare is in a rapid development period, users are uncertain about the security of mHealth services and may worry that their private information and health data might be leaked. It is not difficult to find that when users believe that the adoption of mHealth services may bring them risk of privacy, it will directly reduce their perception of the usefulness of mHealth services, that is, users’ privacy concerns negatively affect performance expectancy. Based on this claim, this study proposes the following hypothesis:

Hypothesis 11: Users’ privacy concerns negatively influence performance expectancy.

Privacy concerns are a factor that cannot be ignored in the adoption of mHealth services. The negative effect of privacy concerns on users’ intention to adopt mHealth services has been confirmed by many studies (Wiljer et al., 2008; Najaftorkaman et al., 2015). For example, the collection, processing, analysis and storage of personal health date in the use of mHealth services make users worry that third parties may leak their private information, which triggers users’ privacy concerns (Kaushik et al., 2018). Concerns about the leakage of medical information can reduce users’ intention of adoption (Anna and Jouni, 2018). Gagnon et al. (2016a) found that privacy security was one of the main factors hindering the widespread adoption and use of personal e-health systems. At the same time, Guo et al. (2016) also claimed that privacy concerns had a negative effect on users’ intention to adopt mHealth services in their study of the privacy paradox phenomenon in mHealth services. Therefore, this study proposes the following hypothesis:

Hypothesis 12: Users’ privacy concerns have a negative effect on intention to adopt mHealth services.

The use of mHealth services involves a large amount of personal health data including basic personal data, which raises concerns about privacy. Because mobile healthcare is an emerging technology service, users are more sensitive to privacy protection. If users believe that there are privacy and security issues in using mHealth services, they may reject suggestions from others and react negatively to adopt mHealth services (Najaftorkaman et al., 2015; Gagnon et al., 2016b). This is even more obvious in the healthcare context in China. When one thinks that there may be privacy and security issues in the use of mobile healthcare, one may decline or reject the advice from important people around, even doctors, nurses and other professionals, which will significantly weaken the intention to adopt mHealth services (Hsiao and Chen, 2015). Thus, this study proposes the following hypothesis:

Hypothesis 13: Users’ privacy concerns negatively influence subjective norm.

Regarding mHealth services, when users’ privacy concerns are high, their perceived ubiquity of mHealth services will become weaker. Worrying about the leakage of individual private information, they are resistant to mHealth services, which may automatically block the convenience and mobility of mHealth services. Users’ perception of ubiquity in mHealth services is reduced, leading to lower intention of adoption (Kim and Park, 2012; Guo et al., 2016). This study believes that mHealth services are different from other technical services. Because mHealth services are closely related to personal health and personal data, users are more concerned about the privacy issues, which will cause users to automatically block the ubiquity of mHealth service. Consequently, this study proposes the following hypothesis:

Hypothesis 14: Users’ privacy concerns have a negative effect on perceived ubiquity.

**FIGURE 2:**
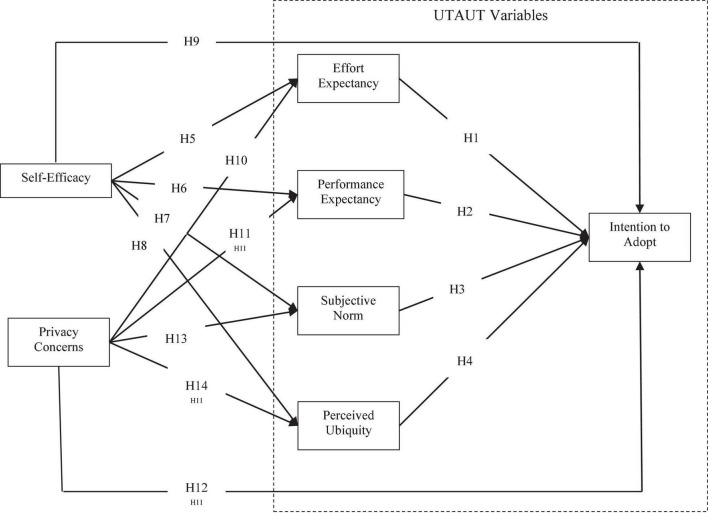
Research model.

By reviewing related theoretical models and literature on users’ intention to adopt mHealth services, this study constructs a model of users’ intention to adopt mHealth services based on the UTAUT model and takes the individual cognitive factors - self-efficacy and privacy concerns – into consideration. The specific conceptual model is shown in Figure 2.

## Research methodology

### Questionnaire development

In order to make the participants fully understand mHealth, the questionnaire started with a detailed introduction to the mHealth services and channels. The first part of the questionnaire set the screening question “Have you ever searched for disease or medical information on the mobile devices?” If the participant answered “No,” the questionnaire was regarded as invalid. The second part consisted of latent variable items. In order to ensure the validity of the measurement, all the items were adapted from mature scales from home and abroad and modified to make them relevant to the context of mHealth in China.

Specifically, effort expectancy and performance expectancy mainly referred to the scale of Venkatesh et al. (2003). Subjective norm, perceived ubiquity, self-efficacy, and privacy concerns were derived from the scales of Lee (2005), Cocosila and Archer (2010), Holden and Karsh (2010), and Guo et al. (2012), and intention to adopt came from the scale of Bhattacherjee (2001a) (see Table 2 for details). All scales were measured using a 7-point Likert-type scale (e.g., 1 = Strongly disagree, 7 = Strongly agree). The third part of the questionnaire included demographic questions and questions about the use of mobile healthcare. Before the final questionnaire was issued, it was first sent to experts in medical-related fields for review and then modified based on their feedback. A pilot testing of questionnaire by 30 respondents was conducted through the platform of Questionnaire Star to ensure the reliability and validity of the questionnaire items, the appropriate difficulty level and rational layout. Participants answered the questions and submitted the questionnaires through the link of Questionnaire Star, a professional online questionnaire survey, evaluation and voting platform. The creator of the questionnaire can download or analyze the data on the Questionnaire Star server (Duan et al., 2020).

**TABLE 2:** Measuring variables and indicators.

Variable	Item	Measurement indicators	Source
Effort Expectancy (EE)	EE1	mHealth operation is simple and easy to understand	Venkatesh et al., 2003
	EE2	I can easily learn to use mHealth	
	EE3	I can independently operate smartphone to obtain mHealth services	
	EE4	It is easy for me to become proficient with mHealth services	
	EE5	Overall, mHealth is easy to learn and use	
Performance Expectancy (PE)	PE1	mHealth provides me with valuable information resources	Venkatesh et al., 2003
	PE2	mHealth can provide me with timely medical information services	
	PE3	mHealth can reduce my queuing and registration time and improve the efficiency of seeing a doctor	
	PE4	mHealth has less time and space constraints, which increases the convenience of life	
	PE5	Overall, mHealth is helpful to my life	
Subjective Norm (SN)	SN1	If my friends, classmates or colleagues use mHealth, I will also use it	Holden and Karsh, 2010
	SN2	If family members and relatives use mHealth, I will also use it	
	SN3	The suggestion of doctors, nurses and other medical professionals will affect my use of mHealth	
	SN4	If a family member who was in poor health for a long time, I would be more likely to use mHealth	
	SN5	When most people use mHealth or mHealth becomes a mainstream, I will also use it	
Perceived Ubiquity (PUB)	PUB1	I can use mHealth at any time	Lee, 2005
	PUB2	I can use mHealth anywhere	
	PUB3	mHealth treatment allows me to seek medical treatment anytime and anywhere, which is very convenient	
Self-Efficacy (SE)	SE1	I can learn how to use mHealth	Guo et al., 2012; Hsieh et al., 2017
	SE2	I am confident that I can skillfully use mHealth	
	SE3	I can meet my medical needs through mHealth	
	SE4	I’m confident in being able to use mHealth independently	
	SE5	I can confidently handle common operational problems when using mobile medical care	
Privacy Concerns (PC)	PC1	mHealth cannot guarantee the confidentiality of users’ personal health information	Cocosila and Archer, 2010
	PC2	Using mHealth may result in misappropriation of personal privacy information	
	PC3	Personal information may be obtained/abused/disseminated by criminals when using mHealth	
	PC4	I’m concerned about personal information leakage when using mHealth to consult more sensitive health issues	
	PC5	If I use mHealth, others may control my health information	
Intention to Adopt (IA)	UI1	When I have related needs, I will choose to use mHealth	Davis, 1989; Taylor and Todd, 1995b; Bhattacherjee, 2001b
	UI2	If mHealth brings convenience to me, I’m willing to continue using it	
	UI3	I’m willing to understand or use mHealth	
	UI4	I’m willing to use mHealth when I face some diseases or health problems	
	UI5	I plan to use mHealth services regularly	

### Data collection

This study used a combination of online and on paper survey to collect empirical data. In the first round, the electronic questionnaire was designed on the questionnaire star website. The questionnaire links were released through instant messaging tools, such as WeChat and QQ, and the secondary release was carried out in a snowball manner. A total of 447 questionnaires were collected. In the second round, 100 paper questionnaires were randomly distributed around a large hospital in China, and 100 questionnaires were recovered. A total of 547 questionnaires were collected in the two rounds, of which 95 questionnaires were excluded due to “no intention to use mHealth,” and 48 samples with the same choices for the items in one construct, 2 samples with the same answers completely, and 16 samples with the mean value greater than or less than 2 SD were deleted (Hair et al., 2014). A total of 386 valid questionnaires were retained, with a valid rate of 70.6%. Among the participants in this survey, there were 204 females, accounting for 52.8%, and 182 males, accounting for 47.2%, with a reasonable gender ratio. In terms of education background, the majority of participants were undergraduates, accounting for about 65%, and nearly 20% of participants had a master’s degree or above. It can be seen that more than 80% of participants have higher education background, indicating that the participants have the ability to effectively fill in the questionnaire, which ensures the effectiveness of data collection to a certain extent. The specific descriptive statistics are listed in Table 3.

**TABLE 3:** Demographic information of the sample (*N* = 386).

Demographics		Frequency	Percentage
Sex	Male	182	47.2
	Female	204	52.8
Age	18–30	161	41.7
	31–40	133	34.5
	41–55	84	21.8
	Older than 56	8	2.1
Education background	Junior high school and below	22	5.7
	High school/vocational school/technical secondary school/Junior College	49	12.7
	Bachelor degree	250	64.8
	Master degree and above	65	16.8
Profession	White-collar workers (state-owned/foreign/private/public institutions)	132	34.2
	Civil servant	21	5.4
	Student	88	22.8
	Individual/private owners	35	9.1
	Freelancer	58	15.0
	Medical worker	26	6.7
	Unemployed	15	3.9
	Others	11	2.8

From the questions about the usage of mHealth services (Table 4), 87.3% of participants have used mHealth services, and nearly 80% of participants have used mHealth services more than twice, indicating that most of the participants have the experience of using mHealth services. They can better fill out the questionnaire based on their personal experience, thus ensuring the accuracy and reliability of the data. The channels through which the participants mainly use mHealth services include the hospital’s official website, WeChat public account and mini programs, accounting for 89.6%. The mHealth services that the participants mainly engage in are making appointment, viewing department and doctor information, and checking about queuing and calling information. In addition, 10.4% of participants used mHealth services before but no longer use it now. The important reasons for participants to stop using mHealth are “No habit of using mHealth services” and “Unguaranteed professionalism and reliability of information,” as shown in Table 5.

**TABLE 4:** Usage of mHealth.

Use features	Category	Frequency	Percentage
Use experience	Used	297	76.9
	Used to use, not use anymore	40	10.4
	Never used	49	12.7
Usage count	1–2 times	66	22.2
	3–4 times	95	32.0
	5–6 times	50	16.8
	More than 6 times	86	29.0

**TABLE 5:** The usage characteristics of mHealth.

Category		Response		Perc of cases (*N* = 386)
		Number	Perc	
Ways to use mHealth (multiple options)	Hospital’s Official Website/Weibo/WeChat Official Account/ Mini Program	266	42.0%	89.6%
	Alipay service window	116	18.3%	39.1%
	Provincial and municipal medical platforms or App	118	18.6%	39.7%
	App for medical consultation (such as: Ping An Good Doctor, Chunyu Doctor)	83	13.1%	27.9%
	Pharmaceutical e-commerce App (such as: 1 Yaowang, Dingdang Kuaiyao) Others	38 12	6.0% 1.9%	12.8% 4%
Use of mHealth services (multiple options)	Making an appointment with a doctor	264	29.9%	88.9%
	Acquiring queuing information	131	14.9%	44.1%
	Intelligent guidance (guide for users to register accurately)	99	11.2%	33.3%
	Visit navigation	104	11.8%	35.0%
	Viewing department and doctor information	146	16.6%	49.2%
	Retrieving medical knowledge	126	14.3%	42.4%
	Others	12	1.4%	4.0%
Reasons for not using it mHealth now (multiple options)	No practical benefit	8	8.9%	19.5%
	No habit of using mHealth	21	23.3%	51.2%
	Unguaranteed professionalism and reliability of information	17	18.9%	41.5%
	Limited functionality	12	13.3%	29.3%
	Cumbersome registration process	13	14.4%	31.7%
	Too many Apps to choose from	14	15.6%	34.1%
	Others	5	5.6%	12.2%

## Results

### Data analysis

To test the reliability and validity of the data, SPSS 25.0 and AMOS 24.0 were used in this study. At the same time, the least squares PLS structural equation model was used to test the posited hypotheses. The proposed model was revised according to the path analysis results to construct the final revised model of intention to adopt mHealth services.

In addition, the Harman single factor test was used to estimate the Common Source Bias (Harris and Mossholder, 1996). This systematic error will make the measurement results deviated from the facts due to the characteristics of subjects, context of items and the single or similar data sources. Podsakoff et al. (2003) suggested that unrotated principal component analysis could be performed on all items at the same time. If no unique factor is formed, the influence of homology bias is insignificant. The test results show that seven factors with eigenvalues greater than 1 were formed without rotation, and the first principal component obtained was 39.74% < 50% (Hair et al., 2007), indicating that there is no serious problem of homologous bias in this study.

### Reliability and validity test

Table 6 presents the standardized factor loadings, AVE values, CR values and Cronbach’ a coefficient for each latent construct. Confirmatory factor analysis (CFA) was performed on 33 items of latent constructs. The results show that the CFA model fitting index is χ^2^ = 1274.863, d.f. = 474, χ^2^/d.f. = 2.690, GFI = 0.825, CFI = 0.919, NFI = 0.878, IFI = 0.920, RMSEA = 0.066, indicating the model has a satisfactory fit. The standard loading coefficients of all factors were between 0.538 and 0.922, which met the validity requirements. The average extraction variance (AVE) value was greater than 0.5, and the CR value and Cronbach’ a value were greater than 0.8. From the above indicators, it can be concluded that the questionnaire meets the requirements of internal consistency reliability and has good convergent validity.

**TABLE 6:** Item loadings, AVE, composite reliabilities, and alpha.

Variable	Item	Loading	AVE	CR	Cronbach’ α
Effort Expectancy (EE)	EE1	0.770	0.691	0.918	0.917
	EE2	0.861			
	EE3	0.831			
	EE4	0.858			
	EE5	0.834			
Performance Expectancy (PE)	PE1	0.747	0.574	0.871	0.868
	PE2	0.786			
	PE3	0.738			
	PE4	0.798			
	PE5	0.716			
Subjective Norm (SN)	SN1	0.821	0.535	0.849	0.847
	SN2	0.851			
	SN3	0.538			
	SN4	0.685			
	SN5	0.718			
Perceived Ubiquity (PUB)	PUB1	0.922	0.788	0.917	0.915
	PUB2	0.916			
	PUB3	0.821			
Self-Efficacy (SE)	SE1	0.759	0.696	0.920	0.916
	SE2	0.873			
	SE3	0.858			
	SE4	0.893			
	SE5	0.781			
Privacy Concerns (PC)	PC1	0.798	0.726	0.930	0.929
	PC2	0.893			
	PC3	0.895			
	PC4	0.846			
	PC5	0.825			
Intention to Adopt (IA)	UI1	0.811	0.688	0.917	0.906
	UI2	0.862			
	UI3	0.842			
	UI4	0.859			
	UI5	0.770			

The correlations between constructs are presented in Table 7. Except that the correlation coefficient between performance expectancy and subjective norm was slightly higher than the square root of AVE of subjective norm, the square root of AVE of other constructs was significantly larger than the correlation coefficient between this construct and other constructs, indicating that the measurement model had satisfactory discriminant validity.

**TABLE 7:** Correlations for latent variables and the square root of AVE.

Variable	IA	PC	SE	SN	PE	EE	PUB
IA	**0.830**						
PC	0.049	**0.852**					
SE	0.615	0.030	**0.835**				
SN	0.697	0.048	0.580	**0.731**			
PE	0.638	–0.031	0.612	0.764	**0.758**		
EE	0.524	0.030	0.657	0.570	0.721	**0.831**	
PUB	0.577	–0.062	0.691	0.614	0.587	0.579	**0.888**

IA, intention to adopt; PC, privacy concerns; SE, self-efficacy; SN, subjective norm; PE, performance expectancy; EE, effort expectancy; PUB, perceived ubiquity. The bolded values are the square root of AVE.

### Hypothesis testing and model revision

**FIGURE 3:**
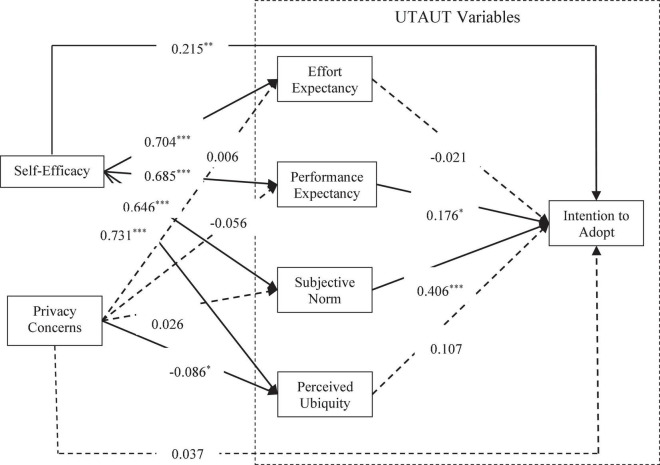
The model of mHealth users’ intention to adopt before correction (standardized path coefficient). *p < 0.05, ^**^p < 0.01, ^***^p < 0.001. The dotted line indicates that the path relationship is insignificant.

AMOS24.0 was used for the path analysis of the structural equation model. The path coefficients and significance levels are shown in Figure 3, and the fitting degree of the model before revision is presented in Table 8. According to the data before revision in Table 8, the overall fitting index of the structural model was poor, so the original hypothetical model was revised accordingly (Whittaker, 2011). In order to improve the fitting degree of the structural model, the model was revised according to Modification Indices in AMOS24.0. The model was revised with the principle of “modification with the highest parameters at a time” (Whittaker, 2011, pp. 694–701). The revised model is shown in Figure 3, and the overall fitting index after the model revision is shown in Table 8. The fitting indices after the model revision were all within the reference value range, so the revised model was acceptable.

**TABLE 8:** Fitting index before and after model correction.

Fit index	χ^2^	df	χ^2^/df	GFI	AGFI	CFI	IFI	RMSEA
Reference	N/A	N/A	≤3	≥0.80	≥0.80	≥0.90	≥0.90	≤0.08
Before correction	1487.573	480	3.099	0.799	0.766	0.898	0.899	0.074
After correction	692.082	286	2.42	0.872	0.843	0.943	0.944	0.061

**FIGURE 4:**
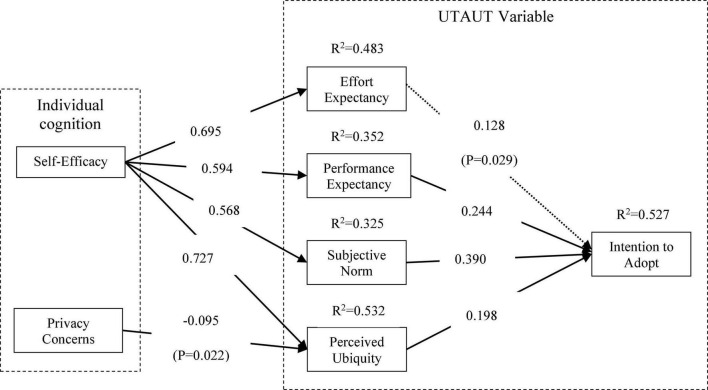
The model of mHealth users’ intention to adopt after correction (standardized path coefficient). *p* < 0.05, *p* < 0.01, *p* < 0.001.

Based on the revised model, we examined the effects of self-efficacy, privacy concerns, effort expectancy, performance expectancy, subjective norm, and perceived service ubiquity on intention to adopt mHealth services. The standardized path coefficients were obtained with a maximum likelihood estimation (Figure 4). It can be seen from the significance of the standardized path coefficient that the four related constructs of the UTAUT model positively and significantly influenced the participants’ intention to adopt mHealth services. Self-efficacy had a significantly positive effect on users’ intention to adopt through effort expectancy, performance expectancy, subjective norm and perceived ubiquity. Privacy concerns only negatively influenced perceived ubiquity, but had no significant effect on effort expectancy, performance expectancy and subjective norm. In addition, the SMCs of effort expectancy, performance expectancy, subjective norm, perceived ubiquity, and intention to adopt were 0.483, 0.352, 0.325, 0.532, and 0.527, respectively, indicating that the model had high explanatory power.

## Discussion

This study aims to investigate users’ intention to adopt mHealth services based on the UTAUT theoretical model. Results from the data analysis provide support for our proposed theoretical model.

First, by analyzing the standardized path coefficients of the original hypothetical model, it can be seen that the main factors affecting users’ intention to adopt mHealth are subjective norm, self-efficacy and performance expectancy. Min et al. (2008); Zhang et al. (2017), Nunes et al. (2019), and Qian and Niu (2019) have all confirmed that these variables have a significant influence on users’ intention to adopt in the fields of mHealth App, mobile payment, mobile shopping and other information systems. In the context of mHealth services, the conclusions of previous studies still hold.

Second, the original hypotheses H2, H3, H5, H6, H7, H8, and H14 are strongly supported both before and after the revision of the model. Both performance expectancy and subjective norm significantly and positively affect users’ intention to adopt mHealth services, among which subjective norm has a greater impact on intention to adopt (β = 0.406, *P* < 0.001), followed by performance expectancy (β = 0.176, *P* < 0.05), indicating that mHealth services indeed increase the convenience of life and increase the willingness of users to use them. In addition, self-efficacy has a significant positive effect on perceived ubiquitous (β = 0.731, *P* < 0.001), effort expectancy (β = 0.704, *P* < 0.001), performance expectancy (β = 0.685, *P* < 0.001) and subjective norm (β = 0.685, *P* < 0.001), indicating that self-efficacy can comprehensively improve users’ perception of mHealth services, and thus improve their willingness to use mHealth services. Privacy concerns have a significantly negative impact on perceived ubiquity (β = –0.086, *P* < 0.05). The higher the user’s concerns about personal privacy, the more likely they are to seek medical treatment through traditional medical service channels, that is, the perceived ubiquity of mHealth services is weakened.

Third, the hypotheses H1, H4, H10, H11, H12, and H13 are not supported in the model before revision, that is, the user’s effort expectancy, privacy concerns and perceived service ubiquity have insignificant effect on intention to adopt mHealth services, while privacy concerns have insignificant impact on effort expectancy, performance expectancy and subjective norm. Of mHealth service users, the youth accounts for more than 70%. They often come into contact with various mobile Apps and have good information literacy. When using mHealth services, they can deal with common operational problems independently. Therefore, effort expectancy and perceived ubiquity do not have significant effect on intention of adoption. In addition, the reason for the insignificant effect of privacy concerns is probably that the young and middle-aged respondents in the sample, who constituted more than half of the mHealth users, were more ready to accept new things and preferred to adopt more convenient mHealth service. Because the youth are less sensitive to personal privacy, and most of the mHealth service channels used by them are highly authoritative platforms such as hospital official websites, WeChat public accounts, and provincial and municipal medical platforms, which shows that information and services are more trustworthy, privacy concerns do not have a significant effect on users’ intention to adopt mHealth services.

Fourth, in the revised users’ intention to adopt mHealth service model, self-efficacy significantly and positively affects users’ effort expectancy (β = 0.695, *P* < 0.001), performance expectancy (β = 0.594, *P* < 0.001), subjective norm (β = 0.568, *P* < 0.001) and perceived ubiquity (β = 0.727, *P* < 0.001). The stronger the user’s self-efficacy, the stronger the intention to adopt mHealth services. For the four variables in the UTAUT model, effort expectancy (β = 0.128, *P* < 0.05), performance expectancy (β = 0.244, *P* < 0.001), subjective norm (β = 0.390, *P* < 0.001) and perceived ubiquity (β = 0.198, *P* < 0.001) can significantly and positively influence users’ intention to adopt mHealth services. Studies by Venkatesh et al. (2003) have verified that effort expectancy and performance expectancy significantly affect users’ intention to adopt. Zhu et al. (2020) also confirmed that subjective norm and perceived ubiquity had a positive effect on users’ intention of adoption. It is easy to find that the conclusions of previous studies still hold in the context of mHealth services.

### Implications for theory

Compared with the existing literature, this paper focuses on the user’s individual cognitive factors and uses the UTAUT theory to analyze the influence of the user’s self-efficacy and privacy concerns on the intention to adopt mHealth services, providing several implications for theory. On the one hand, creatively building an adoption model, this study provides more comprehensive prediction and explanation of users’ adoption behavior of mHealth services by introducing self-efficacy and privacy concerns into the UTAUT model. On the other hand, most of the existing studies focus on the external characteristics, system design, technical environment and usage context of mHealth and many other factors (Chen et al., 2018; Nadal et al., 2020; Wu et al., 2020), but few studies have analyzed the influence of user’s subjective perception on adoption behavior. This paper expands the research perspective of users’ adoption behavior of mHealth service by examining individual cognitive factors and comprehensively considering the direct or indirect effects of users’ self-efficacy and privacy concerns on the intention of adoption.

### Implications for practice

In China’s COVID-19 environment, mHealth services help solve many problems of users’ access to medical care in an epidemic control state. Through mobile terminal devices, users can easily and quickly access various medical services, which reduces the mobility of people to a certain extent and meets the urgent need for epidemic prevention and control. This study conducts an in-depth investigation of users’ intention to adopt mHealth services in China, which helps domestic and foreign mHealth service providers and developers to better optimize the design of mHealth services while enhancing users’ intention to adopt them.

First, the finding in this study suggests that users’ self-efficacy be enhanced by optimizing product design. The stronger the users’ self-efficacy is, the more confident they are in using mHealth services, and the stronger intention they will have to adopt mHealth services. Service providers should optimize product design and user experience, such as providing one-click functional services, simplifying the operation of different sections and improving the ease of use of the interface, so as to enhance users’ confidence in using mHealth services. At the same time, appropriate education on how to use mHealth services should be carried out to improve users’ ability to retrieve, obtain and use medical information or services.

Second, it is desirable that subjective norm should be enhanced by strengthening word-of-mouth communication and recommendation from opinion leaders. The platform should increase the publicity and promotion of typical service cases, paying attention to word-of-mouth communication. The key opinion leaders and experts in the medical field should appropriately recommend mHealth services to users, further expanding the effect of the subjective norm. In addition, service providers should make full use of the trust that users have for the hospitals and doctors to facilitate the adoption of mHealth services of potential users.

Third, it is suggested that the level of privacy security management should be improved in technology, legislation and supervision. It is very important to promote people’s awareness of privacy protection. The service providers should pay close attention to the technical development of privacy protection and make up for the privacy loopholes in the information system, preventing privacy leakage caused by improper technical operation or lack of morality. In addition, the supervision department should strengthen the supervision of mHealth services, formulate relevant laws and regulations, and strengthen the risk assessment and supervision of the operation of mHealth service providers, effectively ensuring the security of users’ private information.

Fourth, the study also implies that the quality of mHealth services should be improved by enhancing the ease of use of the system. The service providers should make effort to simplify the operation interface and operation process, effectively reducing the cost of use. The platform services should be enriched, and the information resources should be updated in time. The users’ inquiries, registration and queuing information should be responded to in a timely manner. In addition, the intelligent recommendations should be added on the medical platform. Personalized recommendations based on full user history, recent activity, or consultation record should be enhanced so that the user can enjoy a better experience and have a sense of acquisition.

Fifth, it is reasonable for service provider to enhance users’ perceived ubiquity by increasing the publicity of the service ubiquity, such as being free from time and space constraints, real-time interaction, convenience and flexibility. The more people know about the convenience that mHealth services bring, the stronger intention of adoption people will have for such services. The platform can also connect users’ hospital treatment data and mHealth service data with smart wearable devices, allowing users to enjoy personalized medical services anytime, anywhere.

### Implications for society

Currently, countries around the world are facing healthcare issues, such as imbalance of healthcare resources and healthcare reform, to varying degrees. UNSDGs are 17 global development goals set by the United Nations to guide global social, economic and environmental development from 2015 to 2030. No. 3 of UNSDGs is ensuring healthy lifestyles and promoting well-being for all at all ages. mHealth services can effectively address the problem of unequal distribution of healthcare resources, improve healthcare coverage, access to medical information, services and skills, as well as promote positive changes in health behaviors, such as in the prevention of emergency and chronic diseases. This study investigates the factors influencing mHealth users’ intention to adopt mHealth by combining the characteristics of mHealth services and users’ individual cognitive factors. The findings can help mHealth service providers provide accurate and effective mHealth services to users, continuously improving users’ access experience and usage stickiness. More importantly, the results of the study will facilitate mHealth services to be widely recognized and used, promoting the sustainable development of mHealth services.

## Conclusion

This study created an integrated model to explain the determinants of users’ intention to adopt toward using mHealth service at the individual cognitive level, extending the UTAUT model by introducing two prediction variables of self-efficacy and privacy concerns. Data were collected from 386 domestic users in China with experiences of using mHealth services. The results reveal that self-efficacy was a key factor that significantly influenced users’ intention to adopt mHealth services. In addition, this study also exhibited that effort expectancy, performance expectancy, subjective norm, and perceived ubiquity all positively influenced users’ intention to adopt. Finally, privacy concerns only had a significantly negative effect on perceived ubiquity, while their effects on effort expectancy, performance expectancy, subjective norm, and intention to adopt were not significant. This study has demonstrated its values to the mHealth service providers by explaining the role of individual perceptions in the decision process of the adoption.

This study also has certain limitations. Firstly, it only studies the user’s intention of adoption, but the intention to adopt is not equal to the behavior of adoption (King and He, 2006). In the future, new variables will be further introduced to explore the user’s intention of adoption and intention of continuous use. Secondly, the questionnaire survey is mainly conducted among 18–40 years old young and middle-aged groups, which cannot fully reflect the adoption behaviors of different age groups. Finally, the scope of mHealth services in the survey is somewhat broad, and users mainly use mHealth services for making appointment, which to a certain extent dilutes or even weakens the effect of privacy concerns on intention of adoption. Therefore, in future research, the samples of the questionnaire survey should be expanded, such as users over the age of 40 such as patients, women and the elderly, and a stratified sampling survey should be conducted in different age groups. The method of “scenario experiment + questionnaire survey” can be used to more comprehensively and empirically investigate the use of mHealth services among different user groups.

## Data availability statement

The raw data supporting the conclusions of this article will be made available by the authors, without undue reservation.

## Ethics statement

Ethical review and approval was not required for the study on human participants in accordance with the local legislation and institutional requirements. The patients/participants provided their written informed consent to participate in this study.

## Author contributions

YL contributed to the study design and wrote the manuscript drafts. CL supervised the study and provided suggestions for the revision of the manuscript drafts. XL contributed to the analysis of the data. GZ and JS provided some reviews on the manuscript. All authors contributed to the article and approved the submitted version.
