# Supplementary material for: Adoption of mobile health services using the unified theory of acceptance and use of technology model: Self-efficacy and privacy concerns
Source: Front Psychol. 2022 Aug 11;13:944976. doi: 10.3389/fpsyg.2022.944976 (PMC9403893; doi:10.3389/fpsyg.2022.944976)
Supplement: Supplementary file 1 [file Data_Sheet_1.doc]

**SUPPLEMENTARY MATERIAL**

**Questionnaire on the intention to adopt mobile health services**

| **Part I Screening Items**   |  | | | | | --- | --- | --- | --- | | 1. Have you ever searched for disease or medical information on the mobile devices? | | | | | A.Yes | | | | | B.No (Please stop answering!) |  |  |  | | | | | | | | | | | | |
| --- | --- | --- | --- | --- | --- | --- | --- | --- | --- | --- | --- | --- | --- | --- | --- | --- | --- | --- | --- | --- | --- | --- | --- | --- | --- | --- | --- |
|
| **Part II Measurement items** | | | | | | | | | | | |
| If you agree with the following point of view, please mark “√” directly on the corresponding scale. | | | | | | | | | | | |
| 1 = Strongly disagree, 2 = Disagree, 3 = A little disagree, 4 = Neutrality, 5 = A little agree, 6 = Agree, 7 = Strongly agree. | | | | | | | | | | | |
| Item | Strongly disagree → Strongly agree | | | | | | | | | | |
| 1. mHealth operation is simple and easy to understand | | 1 | | | 2 | 3 | 4 | 5 | 6 | 7 |  |
| 2. I can easily learn to use mHealth | | 1 | | | 2 | 3 | 4 | 5 | 6 | 7 |  |
| 3. I can independently operate smartphone to obtain mHealth services | | 1 | | | 2 | 3 | 4 | 5 | 6 | 7 |  |
| 4. It is easy for me to become proficient with mHealth services | | 1 | | | 2 | 3 | 4 | 5 | 6 | 7 |  |
| 5. Overall, mHealth is easy to learn and use | | 1 | | | 2 | 3 | 4 | 5 | 6 | 7 |  |
| 6. mHealth provides me with valuable information resources | | 1 | | | 2 | 3 | 4 | 5 | 6 | 7 |  |
| 7. mHealth can provide me with timely medical information services | | 1 | | | 2 | 3 | 4 | 5 | 6 | 7 |  |
| 8. mHealth can reduce my queuing and registration time and improve the efficiency | | | 1 | | 2 | 3 | 4 | 5 | 6 | 7 |  |
| 9. mHealth has less time and space constraints, which increases the convenience of life | | 1 | | | 2 | 3 | 4 | 5 | 6 | 7 |  |
| 10. Overall, mHealth is helpful to my life | | 1 | | | 2 | 3 | 4 | 5 | 6 | 7 |  |
| 11. If my friends, classmates or colleagues use mHealth, I will also use it | | 1 | | | 2 | 3 | 4 | 5 | 6 | 7 |  |
| 12. If family members and relatives use mHealth, I will also use it | | 1 | | | 2 | 3 | 4 | 5 | 6 | 7 |  |
| 13. The suggestion of medical professionals will affect my use of mHealth | | 1 | | | 2 | 3 | 4 | 5 | 6 | 7 |  |
| 14. If a family member who was in poor health, I would be more likely to use mHealth | | | | 1 | 2 | 3 | 4 | 5 | 6 | 7 |  |
| 15. When most people use mHealth, I will also use it | | 1 | | | 2 | 3 | 4 | 5 | 6 | 7 |  |
| 16. I can use mHealth at any time | | 1 | | | 2 | 3 | 4 | 5 | 6 | 7 |  |
| 17. I can use mHealth anywhere | | 1 | | | 2 | 3 | 4 | 5 | 6 | 7 |  |
| 18. mHealth allows me to seek medical treatment anytime and anywhere | | 1 | | | 2 | 3 | 4 | 5 | 6 | 7 |  |
| 19. I can learn how to use mHealth | | 1 | | | 2 | 3 | 4 | 5 | 6 | 7 |  |
| 20. I am confident that I can skillfully use mHealth | | 1 | | | 2 | 3 | 4 | 5 | 6 | 7 |  |
| 21. I can meet my medical needs through mHealth | | 1 | | | 2 | 3 | 4 | 5 | 6 | 7 |  |
| 22. I’m confident in being able to use mHealth independently | | 1 | | | 2 | 3 | 4 | 5 | 6 | 7 |  |
| 23. I can confidently handle common operational problems when using mHealth | | 1 | | | 2 | 3 | 4 | 5 | 6 | 7 |  |
| 24. mHealth cannot guarantee the confidentiality of users’ personal health information | | 1 | | | 2 | 3 | 4 | 5 | 6 | 7 |  |
| 25. Using mHealth may result in misappropriation of personal privacy information | | 1 | | | 2 | 3 | 4 | 5 | 6 | 7 |  |
| 26. Personal information may be disseminated by criminals when using mHealth | | 1 | | | 2 | 3 | 4 | 5 | 6 | 7 |  |
| 27. I’m concerned about personal information leakage when using mHealth to consult more sensitive health issues | | 1 | | | 2 | 3 | 4 | 5 | 6 | 7 |  |
| 28. If I use mHealth, others may control my health information | | 1 | | | 2 | 3 | 4 | 5 | 6 | 7 |  |
| 29. When I have needs, I will choose to use mHealth | | 1 | | | 2 | 3 | 4 | 5 | 6 | 7 |  |
| 30. If mHealth brings convenience to me, I’m willing to continue using it | | 1 | | | 2 | 3 | 4 | 5 | 6 | 7 |  |
| 31. I’m willing to understand or use mHealth | | 1 | | | 2 | 3 | 4 | 5 | 6 | 7 |  |
| 32. I’m willing to use mHealth when I face some diseases or health problems | | 1 | | | 2 | 3 | 4 | 5 | 6 | 7 |  |
| 33. I plan to use mHealth services regularly | | 1 | | | 2 | 3 | 4 | 5 | 6 | 7 |  |

| **Part III Basic information**  **For the following items, please tick “√” in the corresponding choice.** | | | | |
| --- | --- | --- | --- | --- |
| 1. Gender: | | | | |
| A. Male | B. Female |  | |  |
| 2. Age: | | | | |
| A. Younger than 18 | 1 B.18-30 | C.31-40 | |  |
| D.41-55 | E. Older than 56 |  | |  |
| 3. Education background: | | | | |
| A. Junior high school and below | B. High school / vocational school / technical secondary school / Junior College | C. Bachelor degree | D. Master degree and above | |
| 4. Profession: | | | | |
| A. White-collar workers (state-owned / foreign / private / public institutions) | B. Civil servant | C. Student | D. Individual / private owners | |
| E. Freelancer | F. Medical worker | G. Unemployed | H. Others | |
| 5. Experience of using mHealth: | | | | |
| A. Being use | B. Used in past, not use anymore  (Please skip to question 9!) | C. Never used (Please complete!) | |  |
| | 6. Ways to use mHealth: (multiple choices are allowed) | | | | | |  | | | | | | | | | --- | --- | --- | --- | --- | --- | --- | --- | --- | --- | --- | --- | --- | --- | | A. Hospital’s Official Website/Weibo/WeChat Official Account/Mini Program | | B. Alipay service window | | C. Provincial and municipal   medical platforms or App | | | | | | |  | |  | | D. App for medical consultation  (such as: Ping An Good Doctor, Chunyu Doctor) | | E. Pharmaceutical e-commerce App (such as: 1 Yaowang, Dingdang Kuaiyao) | | | F. Others | | | |  |  | | | | | 7. Usages of mHealth services: (multiple choices are allowed)) | | | | | |  | | | | | | | | | A. Making an appointment  with a doctor | B. Acquiring queuing information | | C. Intelligent guidance  (guide for users to register accurately) | | | | | D.Visit navigation | | | | | | | E. Viewing department &  doctor information | F. Retrieving medical knowledge | | G. Others | | | |  | | | | |  | |   8. How many times have you used mHealth before? | | | | |
| A.1-2 times | B.3-4 times | C.5-6 times | | D. More than 6 times |
| 9. Reasons for not using mHealth now: (multiple choices are allowed) | | | | |
| A. No practical benefit | B. No habit of using mHealth | C. Unguaranteed professionalism  and reliability of information | | D. Limited functionality |
| E. Cumbersome registration process | F. Too many Apps to choose from | G. Others | |  |
